# Parental involvement and math anxiety: a dual pathway to the math anxiety of children

**DOI:** 10.3389/fpsyg.2026.1792902

**Published:** 2026-07-06

**Authors:** Rui Wang

**Affiliations:** School of Education, Hulunbuir University, Hulunbuir, China

**Keywords:** children’s math anxiety, family environment, moderation, parental involvement, parental math anxiety

## Abstract

**Objective:**

Despite the importance of parental factors in the math anxiety of children, the influence of the interaction between parental involvement and parental math anxiety on the math anxiety of children has not been sufficiently explored. This study is aimed at investigating how parental math anxiety moderates the association of parental involvement with the math anxiety of children.

**Methods:**

Data were collected from 450 seventh-grade students and their parents in China, including parental involvement, parental math anxiety and the math anxiety of children. Process Model 1 was used to perform moderated regression analysis.

**Results:**

Relationship analysis indicated that mother (*r* = −0.15, *p* < 0.01) and father involvement (*r* = −0.18, *p* < 0.01) both showed a significant negative correlation with the math anxiety of children. In addition, parental math anxiety exhibited a significant positive correlation with the math anxiety of children (*r* = 0.29, *p* < 0.01). Mother involvement significantly interacted with parental math anxiety (*β* = −0.12, *p* = 0.017, 95% CI [−0.21, −0.02]), and father involvement also significantly interacted with parental math anxiety (*β* = −0.12, *p* < 0.01, 95% confidence interval (CI) [−0.20, −0.04]). Specifically, the negative relationship of parental involvement with the math anxiety of children was significant only among parents with high (mother involvement: *β* = −0.259, *p* < 0.05; father involvement: *β* = −0.292, *p* < 0.05) rather than low math anxiety (mother: *β* = −0.029, *p* > 0.05; father: *β* = −0.053, *p* > 0.05). Furthermore, gender analysis demonstrated that this moderating effect was significant only for girls instead of boys.

**Conclusion:**

Parental math anxiety moderates the association of parental involvement with the math anxiety of children. Overall, parental involvement is negatively linked to the math anxiety of children (significant main effect). After parental math anxiety was included as a moderator, this negative relationship was present only in the group with high parental math anxiety and was non-significant in the group with low parental math anxiety. This finding poses a challenge to the assumption that “high parental math anxiety is necessarily harmful”. Furthermore, this moderating effect was more pronounced among girls.

## 1 Introduction

From the perspective of many students, mathematics is difficult and out of reach (Fronberg, 2021), which can lead to a situation called math anxiety. Concerning math anxiety, it refers to a negative feeling of people when they face problems related to mathematics (Ashcraft et al., 2007). Harari et al. (2013) stated that an increasing number of students experience math anxiety from elementary school through high school. This anxiety can start as early as preschool (Devine et al., 2018; Dowker et al., 2016). With the growth of children, math anxiety grows like a snowball, which exerts an influence on their achievements and future life choices (Rolison et al., 2020). In school settings, math anxiety can have numerous harmful effects, which cause negative psychological impacts, behavior issues and physical symptoms (Rubinsten et al., 2018), like feeling sick, helpless and anxious. Some people actively avoid math activities. Due to these feelings, students are even more fearful of mathematics (Lucietto et al., 2020). Consequently, they do not practice very often (Ashcraft et al., 2007; Berger et al., 2020). This puts students at a big disadvantage, probably affecting their decision to pursue a career (Wang and Degol, 2017). Therefore, the math anxiety of school students should be taken seriously.

Research has shown that math anxiety has turned into a common psychological phenomenon around the globe (Lau et al., 2022). Previous studies have focused on the influence of math anxiety (Devine et al., 2018) and paid less attention to its causes (Rubinsten et al., 2018). Math anxiety negatively affects the development of children. In recent years, researchers have thus shifted their horizons to explore the mechanisms and interventions shaping math anxiety (Rubinsten et al., 2018). However, understanding the family origins of math anxiety requires situating it within the broader context of parental educational anxiety.

In July of 2021, a series of educational regulations called the “Double Reduction” policy and formally titled “Opinions on Further Reducing the Burden of Homework and Off-Campus Tutoring for Students in Compulsory Education” were introduced in China (General Office of the Central Committee of the Communist Party of China and General Office of the State Council, 2021). The “Double Reduction” policy strictly limits the total amount of homework assigned to pupils in compulsory education and prohibits for-profit academic tutoring off campus. Against the backdrop of this policy, empirical studies have examined the evolution of parental educational anxiety. According to Chen et al. (2022), parental educational anxiety did not subside as a result of the policy, but took on new forms. The gap between parental educational expectations and the actual academic performance of their children emerged as a significant predictor of anxiety levels. Zhang et al. (2025) further noted that socioeconomic factors, including household income, educational expenditure, parents’ own educational attainment, etc., directly influence educational anxiety.

Nevertheless, not all subject-related anxiety is equally related to parental involvement. Math anxiety is particularly noteworthy for the following reasons: it tends to appear earlier than other academic anxieties and usually manifests as early as primary school or even preschool (Devine et al., 2018). Furthermore, it is more closely linked to avoidance behaviors and long-term career choices, particularly in science, technology, engineering and mathematics (STEM) fields (Wang and Degol, 2017). Different from anxiety associated with English or physical education classes, math anxiety can be easily transmitted from parents to children via everyday interactions (Maloney et al., 2015). For these reasons, math anxiety provides an especially illuminating context for examining how subject-specific emotions develop within the family.

Although these studies have deepened the understanding of educational anxiety, little discussion has been held on the role of subject-specific anxiety, like math anxiety, in parent–child interactions up to now. Thus, this study shifts its focus to the field of mathematics to examine how the math anxiety of parents moderates the relationship between their engagement in the math learning and the anxiety of their children. Compared with general educational anxiety, math anxiety features early onset (Devine et al., 2018), cross-cultural prevalence (Lau et al., 2022) and strong intergenerational transmission (Maloney et al., 2015). Hence, while educational anxiety provides a macro-level theoretical context, centering specifically on math anxiety is conducive to revealing subject-specific mechanisms that cannot be captured by the tools for the measurement of general educational anxiety. Within this theoretical framework, the present study examines the influence of parental factors in the family environment on the math anxiety of children. Furthermore, most prior research made a combination of mother and father involvement, while the current study explores them separately to highlight the distinct roles of maternal and paternal involvement.

## 2 Literature review

### 2.1 Parental involvement and the math anxiety of children

It has been shown that parental involvement is a key factor influencing the math anxiety of children (Barger et al., 2019; Maloney et al., 2015; Zhao et al., 2021; Si et al., 2022; Li et al., 2021). In the field of mathematics, a great deal of empirical research has argued that parental involvement may have different effects on the academic performance and affect of children (Ching et al., 2021; Daches Cohen and Rubinsten, 2017; Del Río et al., 2017; Demirtaş and Uygun-Eryurt, 2020; Vanbinst et al., 2020). Depending on involvement quality and style, supportive, warm and low-pressure involvement is inclined to relieve the math anxiety of children and improve their academic motivation, whereas intrusive, controlling or anxiety-driven involvement may increase negative emotions and impair learning confidence (Cheung and Pomerantz, 2011).

In China, several studies have delved into the associations of parental involvement with the academic outcomes and emotions of children. These studies, including those by Luo et al. (2014), Wang (2021) and Pang (2020), have consistently discovered that parental involvement exerts a positive impact on both aspects of children’s schooling. For example, Wang (2021) surveyed 1,086 Chinese junior high school students. It was found that parental involvement and its three sub-constructs all significantly and positively predicted the academic achievements of junior high school students. Moreover, good parent–child relationships, like providing children with a supportive and stable learning environment, contribute to the academic achievements of children. Parent–child relationships play an important moderating role between parental involvement and the academic achievements of children. Additionally, an empirical study by Pang (2020) utilized data from the China Education Tracking Survey (CEPS) between 2014 and 2015, and produced similar results.

Beyond that, Luo et al. (2014) found that parental expectations, support, encouragement and good family-school communication can significantly enhance the academic performance of children. In addition, parents can also help establish a good academic and emotional state in their children by creating a relaxing and enjoyable home learning environment and actively participating in a range of activities organized by the school.

However, research by Fang (2022) reminded that parents may exacerbate the math anxiety of their children when overly intervening in their children’s math learning, especially when having high math anxiety themselves. To be specific, parental math anxiety reduces the academic performance of children. When excessively involved in the math learning of their children, parents with high math anxiety may unintentionally pass on their anxiety about math to their children. This causes their children to experience anxiety and fear of math emotionally.

### 2.2 Moderator role of parental math anxiety

Although a growing amount of research suggests a link between parental involvement and the math anxiety of children, the ways in which parental involvement affects the anxiety of children about math remain poorly understood. Recent studies have emphasized that the math anxiety of parents negatively influences the math learning of their children. Children whose parents experience higher-level math anxiety are more likely to learn less math and worry more about the subject (DiStefano et al., 2020; Linares, 2023). More specifically, a range of specific behaviors can negatively influence parental math anxiety when parents tutor their children in math. Firstly, a lack of self-confidence leads to hesitation and uncertainty in the tutoring process. Secondly, parents will feel a sense of frustration and loss when having difficulty in providing effective study guidance. Thirdly, conflict and stress arise when parents disagree with their children over academic communication. This series of behaviors not only affects the emotions of parents but also creates a stressful learning environment for children, which further exacerbates the anxiety of children.

Understanding the interplay of parental involvement with parental math anxiety is critical to understanding its influence on the math anxiety of children. Emotional contagion theory (Hatfield et al., 1993) shows that people can “catch” each other’s emotions. Research has demonstrated an intergenerational link between parents and children in math anxiety (Vanbinst et al., 2020). Parents with higher-level math anxiety are frequently involved in the math learning of their children and can influence the math anxiety of their children. When parents experience high anxiety levels and excessively get involved in the math activities of their children, their children are inclined to learn less math throughout the school year and experience more math anxiety (Maloney et al., 2015). However, whether this negative pattern applies to a variety of cultures or contexts remains an open question.

### 2.3 Research model

**Figure 1:**
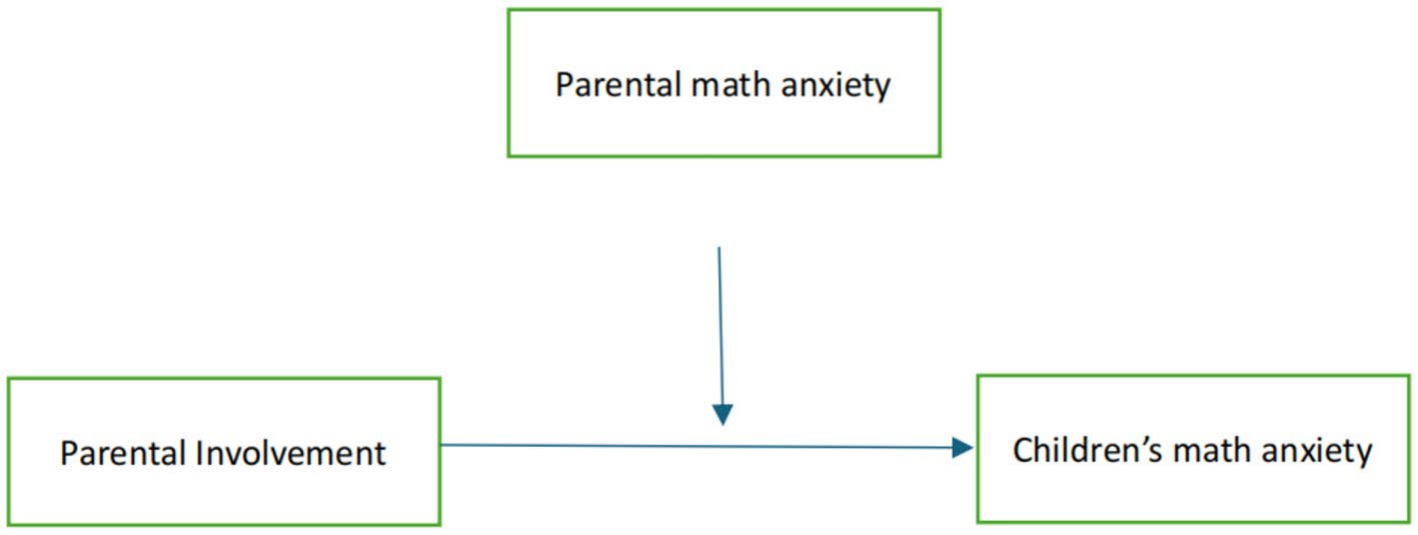
Research modeling with parental math anxiety as a moderator variable.

This study presents a moderation model explaining the association between parental involvement, children’s math anxiety and parental math anxiety. In specific terms, it hypothesizes that parental involvement will substantially reduce the math anxiety of children, while parental math anxiety significantly and positively predicts the math anxiety of children. The current study further explores the moderating role of parental math anxiety in the relationship between parental involvement and the math anxiety of children (see Figure 1). That is, the effect of parental involvement on the math anxiety of children becomes weaker in the case of higher-level parental math anxiety. Ultimately, the present study expects to examine the complex connection between parental involvement, the math anxiety of children and parental math anxiety to provide theoretical support for educators, teachers and parents.

## 3 Method

### 3.1 Participants and procedure

Study participants included 450 seventh-grade students and their parents (fathers or mothers) from three secondary schools in Inner Mongolia, China. Students were aged 11–14, with an average age of 12.82. Of them, 53.3% were girls, and 46.7% were boys, which indicated an equal proportion of participants. Apart from that, about three-quarters of parent participants were mothers (74.7%), while 25.3% were fathers.

To measure parental involvement, parental math anxiety and the math anxiety of children, a paper questionnaire was used to collect data. First, the principals of schools were contacted, and a request letter was sent to seek cooperation. Second, students and their parents offered informed consent to participate before formal testing, which was completed with the consent of students and their parents. Third, the researcher met with the classroom teacher to explain the study’s purpose and promise confidentiality. Fourth, a paper questionnaire was administered to students and their parents during parent-teacher conferences to gather data on students’ math anxiety, perceived parental involvement, students’ attitude toward math, and parental math anxiety. The researcher read a standardized guide aloud during the test. Participants were required to complete the questionnaire independently and return it on the spot at the end of the test. Fifth, completed questionnaires were gleaned and organized. Finally, the data collected were analyzed.

### 3.2 Measures

#### 3.2.1 Parental involvement

In this study, parental involvement refers to the behaviors that parents exhibit to promote the development of their children during mathematics learning of their children. The parental involvement questionnaire developed by Song (2010) was adopted in this study. The questionnaire completed by children reflected their perception of their parents’ level of involvement in their math learning. This questionnaire consisted of two sections: father and mother involvement. It was divided into three dimensions, namely emotional, intellectual and behavioral management involvement, each with 21 questions (see Table 1). In total, 42 questions were included. The first dimension, emotional involvement, means parents’ understanding of their children’s school and learning emotions. The second dimension, intellectual involvement, refers to providing their children with learning materials or activities contributing to learning and intellectual development. The third dimension behavioral management involvement indicates parents’ management and guidance of their children’s learning-related behaviors. Participants rated their agreement with all items on a five-point Likert scale ranging between 1 (*Never*) and 5 (*Always*). The middle score denotes “neutral.” In this study, father and mother involvement had a Cronbach’s alpha of 0.94 and 0.92, respectively.

**Table 1:** Measure of parental involvement.

Parental involvement	Items (numbers)	Examples of items	Alpha (item source)
Emotional involvement	Father:(6) 20,17,21,13,11,16 Mother:(6) 3,17,18,21,20,19	Father: Father gives you encouragement when you do not do well in exams. Mother: Mothers understand your feelings at school.	Father:0.82 Mother:0.83 (Song, 2010)
Intellectual involvement	Father:(9) 4,7,10,1,15,2,19,6,14 Mother:(9) 8,11,6,5,15,1,2,13,14	Father: Father buys you study guides or materials. Mother: Your mother tutoring you in your studies.	Father:0.86 Mother:0.80 (Song, 2010)
Behavioral management involvement	Father:(6) 3,5,12,8,18,9 Mother:(6) 4,12,9,10,7,16	Father: Father manages your time online. Mother: Mother asks you about your homework completion.	Father:0.77 Mother:0.71 (Song, 2010)

#### 3.2.2 Math anxiety of children

In the present study, the math anxiety of children means a negative emotion of tension and anxiety that children experience when tackling math-related problems. Zhang (2017) was chosen for inclusion in this study. The questionnaire comprises 15 items addressing three distinct sub-constructs: math evaluation, learning and problem-solving anxieties (including six, five and four items, respectively). The questionnaire was administered on a five-point Likert scale (1 = *no anxiety*, 5 = *very anxious*). Cronbach’s alpha for this investigation was 0.93.

#### 3.2.3 Parental math anxiety

In this study, parental math anxiety refers to parents’ state of anxiety, worry and fear when they deal with situations requiring mathematical knowledge and skills. The Abbreviated Math Anxiety Scale (AMAS), invented by Hopko et al. (2003), was selected for the current study. It is composed of nine items involving two constructions: learning math (containing five items) and math evaluation anxieties (containing four items). The AMAS has nine items on a five-point Likert scale ranging between 1 (*low anxiety*) and 5 (*high anxiety*). A Cronbach alpha of 0.93 was found in this investigation.

### 3.3 Data analysis

Initially, a common method bias test was conducted to assess potential biases arising from the adoption of self-reported measures. After that, SPSS 23.0 was utilized for computing descriptive statistics and Pearson correlations for parental involvement, parental math anxiety and the math anxiety of children. Next, a moderated effects test was performed using Model 1 in Hayes’s SPSS macro software PROCESS 4.1-SPSS, and standardized continuous variables were employed. To ensure consistent standard error estimates for parameters, the effects were further investigated using bootstrap techniques (Harari et al., 2013). Hayes (2012, 2017) claimed that an effect is considered substantial if the 95% confidence intervals (CIs) for these effects are exclusive of zero, on the basis of bootstrap sampling from a sample of 5,000.

## 4 Results

### 4.1 Common method bias test

To check for bias, the data were analyzed using the Harman one-way test (Podsakoff et al., 2003). The findings show that 13 frequent components had eigenvalues above 1. Zhou and Long (2004) held that the common method bias of this study was not significant since the first common component only explained 17.21% of the variance, which was below the key requirement of 40%.

### 4.2 Correlation analysis and descriptive statistics for variables

The correlation matrix and standard deviation of primary variables used in this investigation are illustrated in Table 2. Significant negative correlations were observed between both father/mother involvement and the math anxiety of children. The math anxiety of children exhibited a significant positive association with parental math anxiety.

**Table 2:** Correlation analysis and descriptive statistics for variables.

Variable	*M*	SD	1	2	3	4
1. Mother involvement	3.84	0.81	1	1	1	1
2. Father involvement	3.39	1.00	0.60**			
3. Children’s math anxiety	2.56	0.95	−0.15**	−0.18**		
4. Parental math anxiety	2.51	0.94	−0.05	−0.05	0.29**	

Dependent variable: children’s math anxiety; M = mean; SD = standard deviation; *N* = 450. **p < 0.01.

### 4.3 Moderating effect analysis

The main impact of parental involvement on the math anxiety of children was tested in Model 1. The results showed that mother (*β* = −0.15, *p* < 0.01) and father involvement (*β* = −0.18, *p* < 0.01) were significant negative predictors, which established a robust baseline model. Parental math anxiety was added as a predictor in Model 2. The main effects became non-significant, while parental math anxiety showed significant positive effects. Interaction terms were introduced in Model 3. Mother involvement × PMA (*β* = −0.116, *p* < 0.05) and father involvement × PMA (*β* = −0.120, *p* < 0.05) were significant, which confirmed that parental math anxiety played a moderating role (see Tables 3, 4).

**Table 3:** Hierarchical regression results (other involvement).

Dependent variable: children’s math anxiety			
Variable	Model 1 β	Model 2 β	Model 3 β
Step 1			
Mother involvement (MI)	−0.15**	0.12	0.12
Step 2			
Parental math anxiety (PMA)	–	0.71**	0.71**
Step 3			
MI × PMA	–	–	−0.12*
*R* ^2^	0.02**	0.10**	0.11**
Δ*R*^2^	0.02**	0.08**	0.01*
*F*	10.82**	24.76**	18.73**

*N* = 450. *p < 0.05, **p < 0.01.

MI, mother involvement; PMA, parental math anxiety; MI × PMA, interaction term.

**Table 4:** Hierarchical regression results (father involvement).

Dependent variable: children’s math anxiety			
Variable	Model 1 β	Model 2 β	Model 3 β
Step 1			
Father involvement (FI)	−0.18**	0.10	0.10
Step 2			
Parental math anxiety (PMA)	–	0.68**	0.68**
Step 3			
FI × PMA	–	–	−0.12**
*R* ^2^	0.03**	0.12**	0.13**
Δ*R*^2^	0.03**	0.09**	0.01**
*F*	15.15**	29.54**	21.77**

*N* = 450. **p < 0.01.

FI, father involvement; PMA, parental math anxiety; FI × PMA, interaction term.

This study focuses on the effects of parental involvement (both father and mother involvement) on the math anxiety of children. Particular emphasis is placed on determining whether parental math anxiety moderates the influence of parental involvement on the math anxiety of children. Process Model 1 was leveraged to analyze the effects of parental involvement and parental math anxiety on the math anxiety of children. The results indicated that mother involvement was significantly negatively associated with the math anxiety of children (*β* = −0.15, *p* < 0.01) before the introduction of the moderating variable.

After the addition of the moderating variable (parental math anxiety), however, the influence of the independent variable (mother involvement) on the dependent variable (children’s math anxiety) shifted to a positive and non-significant correlation (*β* = 0.12, *p* > 0.05), with a 95% CI containing 0 (lower limit of CI (LLCI) = −0.13, upper limit of CI (ULCI) = 0.38). In addition, the interaction of the moderator variable (parental math anxiety) was significant (*β* = −0.12, *p* = 0.017) and its 95% CI excluded 0 (LLCI = −0.21, ULCI = −0.02). This suggests that the influence of mother involvement on the math anxiety of children was moderated by parental math anxiety (see Table 5 for details).

**Table 5:** Moderating impact test (mother involvement).

Dependent variable: children’s math anxiety					
Variables	Coefficient (β)	*p*-value	*t*-value	LLCI	ULCI
MI (X)	0.12	0.34	0.96	−0.13	0.38
PMA (M)	0.71	0.00	3.73	0.34	1.09
MI × PMA (X × M)	−0.12	0.01**	−2.39	−0.21	−0.02
*R*	0.33				
*R* ^2^	0.11				
*F*-value	18.73	0.00			

*N* = 450. *p < 0.05, **p < 0.01.

MI, mother involvement; PMA, parental math anxiety; MI × PMA, interaction term.

Similar results were obtained for the moderating effect of parental math anxiety between father involvement and the math anxiety of children. Initially, father involvement had a significant impact on the math anxiety of children (*β* = −0.18, *p* < 0.01). After the addition of the moderating variable (parental math anxiety) to the model, however, the influence of father involvement on the math anxiety of children changed, and the main impact of father involvement on the math anxiety of children was non-significant (*β* = 0.10, *p* = 0.31), with a 95% CI containing 0 (LLCI = −0.10, ULCI = 0.31). Also, the interaction of the moderator variable (parental math anxiety) was significant (*β* = −0.12, *p* < 0.01), with 95% CIs excluding 0 (LLCI = −0.20, ULCI = −0.04). This result suggests that the correlation between father involvement and the math anxiety of children was moderated by parental math anxiety (see Table 6 for details).

**Table 6:** Moderating impact test (father involvement).

Dependent variable: children’s math anxiety					
Variables	Coefficient (β)	*p*-value	*t*-value	LLCI	ULCI
FI (X)	0.1	0.31	1.01	−0.1	0.31
PMA (M)	0.68	0.00	4.48	0.38	0.97
FI × PMA (X × M)	−0.12	0.005**	−2.82	−0.2	−0.04
*R*	0.36				
*R* ^2^	0.13				
*F*-value	21.77	0.00			

*N* = 450. **p < 0.01. FI, father involvement; PMA, parental math anxiety; FI × PMA, interaction term.

To better understand the moderation effect, simple slope tests were performed. The results are shown in Tables 7, 8. Parental math anxiety was classified into low and high groups based on one standard deviation below and above the mean. For mother involvement, the negative predictive effect on the math anxiety of children was significant in the group with high parental math anxiety (*β* = −0.259, SE = 0.068, 95% CI [−0.393, −0.126]), but non-significant in the group with low parental math anxiety (*β* = −0.029, SE = 0.074, 95% CI [−0.176, 0.118]). Similarly, for father involvement, the negative predictive effect on the math anxiety of children was significant in the group with high parental math anxiety (*β* = −0.292, standard error (SE) = 0.062, 95% CI [−0.415, −0.170]), but non-significant in the group with low parental math anxiety (*β* = −0.053, SE = 0.057, 95% CI [−0.166, 0.059]). These results revealed that only high-level parental math anxiety exerted a significant moderating effect.

**Table 7:** Moderating effects at different levels of parental math anxiety (mother involvement).

IV: FI	β	SE	95%	
			LL	UL
M-1SD	−0.029	0.075	−0.176	0.118
M + 1SD	−0.259	0.068	−0.393	−0.126

MI, mother Involvement; β, standardized coefficient; SE, standard error; CI, confidence interval; M-1SD, low parental math anxiety group; M + 1SD, high parental math anxiety group.

**Table 8:** Moderating effects at different levels of parental math anxiety (father involvement).

IV: FI	β	SE	95%	
			LL	UL
M-1SD	−0.053	0.057	−0.166	0.059
M + 1SD	−0.292	0.062	−0.415	−0.170

FI, father involvement; β, standardized coefficient; SE, standard error; CI, confidence interval; M-1SD, low parental math anxiety group; M + 1SD, high parental math anxiety group.

**Figure 2:**
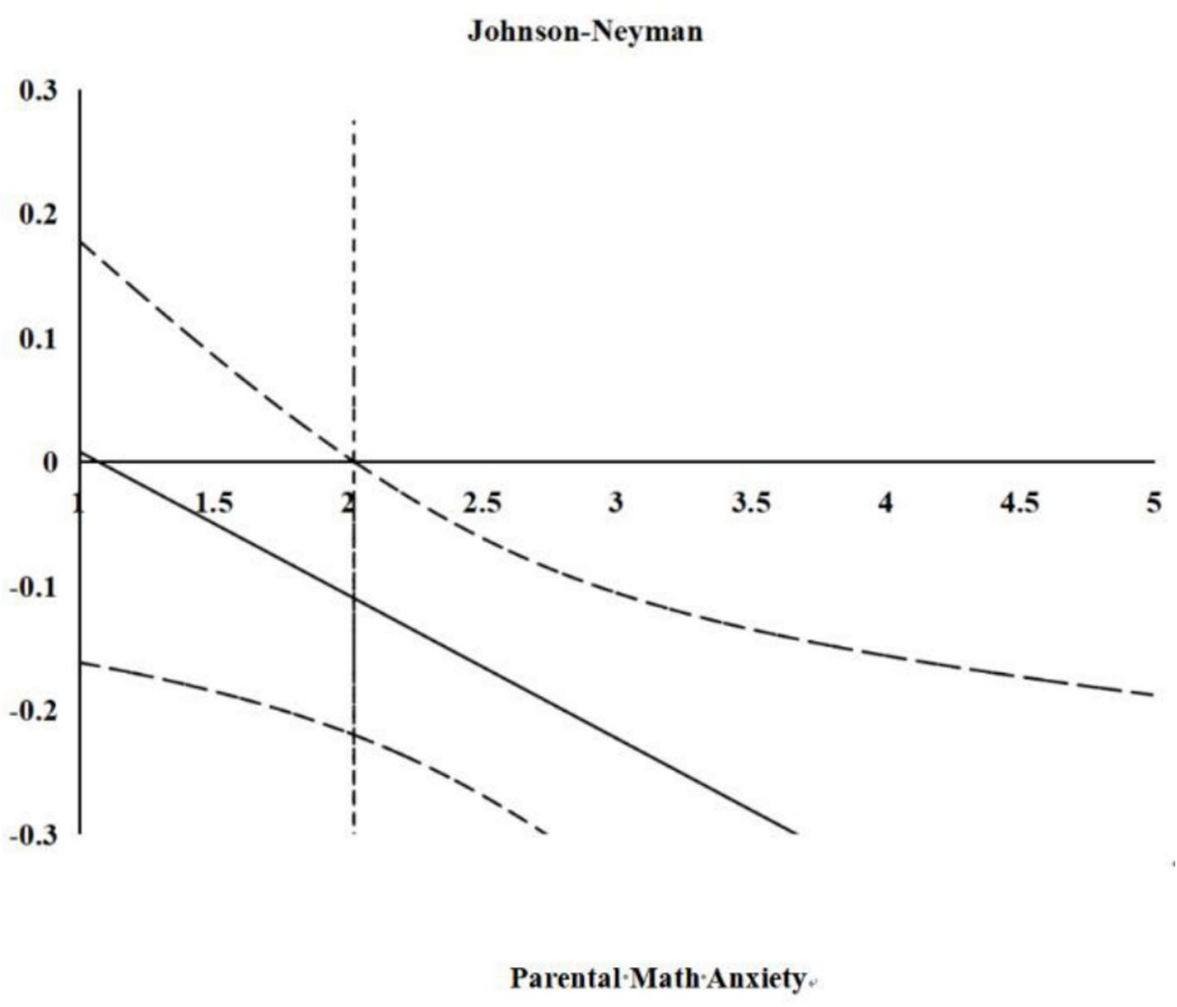
Floodlight analysis of the moderating impact of mother math anxiety. Independent variable: mother involvement; dependent variable: children’s math anxiety.

Furthermore, the moderating impact is a process of continuous change, and the fetch-point method cannot fully reflect the results. As a result, the Johnson-Neyman approach for floodlight analysis was further adopted in this study to present a simple slope change trajectory. As seen in Figure 2, the CI of the regression slope of mother involvement in the math anxiety of children excluded 0 when the raw score of parental math anxiety was above 2.02. This indicates that parental math anxiety exerted a significant moderating effect when parental math anxiety exceeded the above threshold. When the raw score of parental math anxiety was below 2.02, the CI contained 0 points. At this time, the moderating impact of parental math anxiety on parental involvement in the math anxiety of children was non-significant.

**Figure 3:**
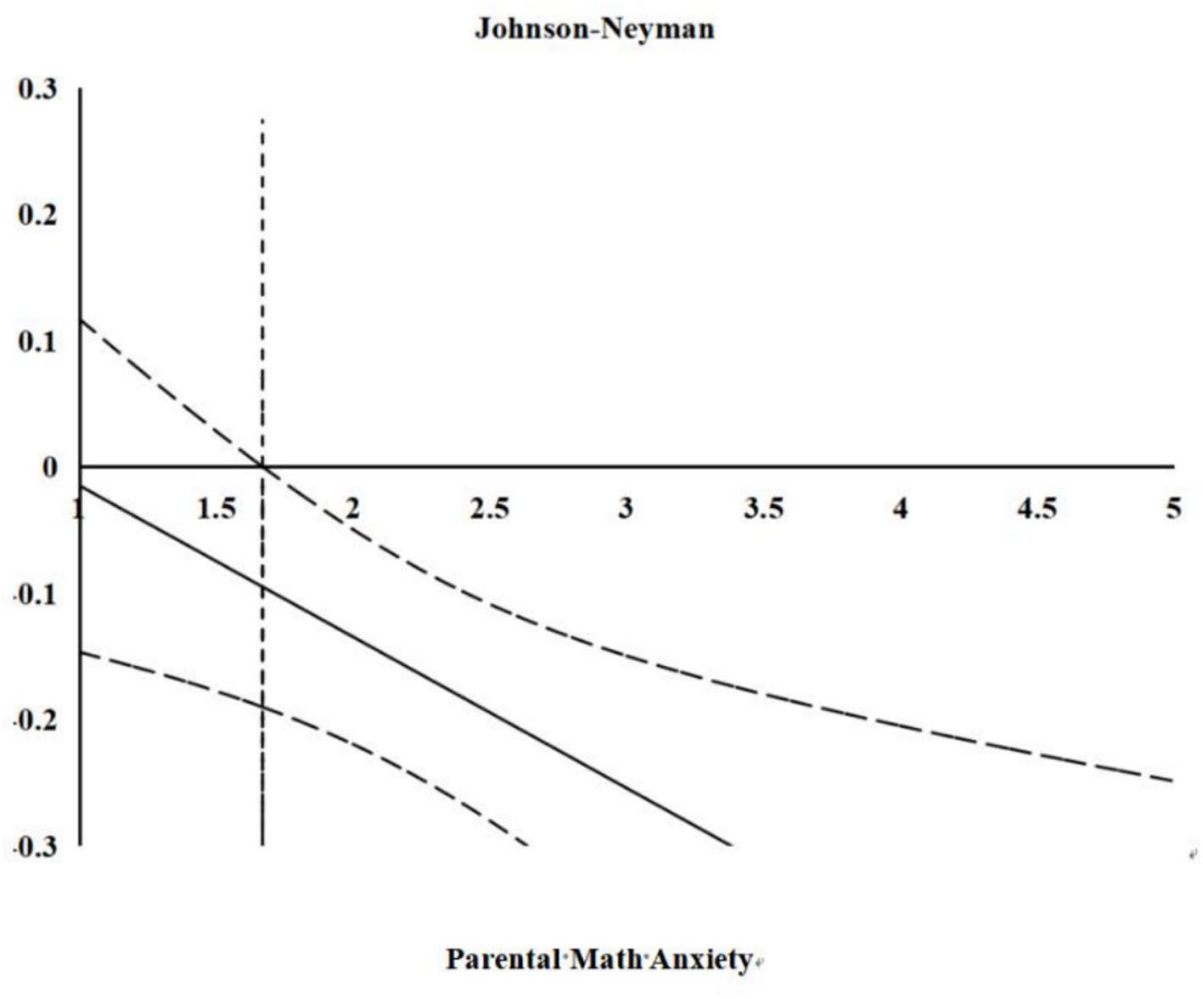
Floodlight analysis of the moderating impact of father math anxiety. Independent variable: father involvement; dependent variable: children’s math anxiety.

As shown in Figure 3, the CI of the regression slope of father involvement affecting the math anxiety of children excluded 0 when parental math anxiety was greater than the critical value of 1.67. This indicates that the moderating impact of parental math anxiety was significant. Meanwhile, when the raw score of parental math anxiety was less than 1.67, the moderating effect of parental math anxiety on the association between the math anxiety of children and parental involvement was non-significant, with a CI of 0. Parental math anxiety did not play a significant moderating role between parental involvement and students’ math anxiety.

### 4.4 Gender differences in the moderating effect of parental math anxiety

Stratified regression analyses were conducted separately for boys (*n* = 201) and girls (*n* = 249). The results demonstrated that the interaction of parental involvement with parental math anxiety showed statistical significance only among girls (maternal involvement × parental math anxiety: *B* = −0.139, *p* = 0.011; father involvement × parental math anxiety: *B* = −0.148, *p* = 0.026), but it was non-significant among boys (mother: *B* = 0.001, *p* = 0.989; father: *B* = −0.055, *p* = 0.318). Therefore, the moderating effect of parental math anxiety is only applicable to girls (see Table 9).

**Table 9:** Hierarchical regression results for the moderating effect of parental math anxiety by child gender.

Gender	*n*	Interaction term	*B*	*p*	95% CI
Boy	201	MI × PMA	0.001	0.9889	[−0.140, 0.160]
Boy	201	FI × PMA	−0.055	0.3180	[−0.165, 0.055]
Girl	249	MI × PMA	−0.139	0.0110	[−0.250, −0.032]
Girl	249	FI × PMA	−0.148	0.0260	[−0.267, −0.029]

MI, mother involvement; FI, father involvement; PMA, parental math anxiety; B, unstandardized coefficient; CI, confidence interval.

## 5 Discussion

### 5.1 Overall findings and the moderating role of parental math anxiety

In this study, the levels of parental involvement and math anxiety in both fathers and mothers were analyzed separately, and the unique influence of parents on the math anxiety of children was revealed. The results reveal the moderating role of parental math anxiety between parental involvement and the math anxiety of children. Specifically, the effect of parental involvement on the math anxiety of children is influenced by the level of parental math anxiety. This means that whether parental math anxiety is present or absent can alter the trend and impact of parental involvement on the math anxiety of children.

Notably, parental math anxiety only played a significant moderating role above a critical threshold (raw score > 2.02 for mother involvement; raw score > 1.67 for father involvement). Below this threshold, parental math anxiety failed to moderate the association of parental involvement with the math anxiety of children. This indicates that the conditional nature of the involvement–anxiety link depends on the math anxiety level of parents. The correlation analysis results verified that parental involvement was significantly negatively correlated with the math anxiety of children (Barger et al., 2019; Zhao et al., 2021). Furthermore, parental math anxiety exhibited a significant positive association with the math anxiety of children. This is a finding consistent with prior research indicating an intergenerational relationship between parents and children in the context of math anxiety (DiStefano et al., 2020; Maloney et al., 2015; Vanbinst et al., 2020).

### 5.2 Conditional protective effect: the role of high parental math anxiety

According to emotional contagion theory (Hatfield et al., 1993), individuals are able to perceive and internalize each other’s emotions during interpersonal communication. In the context of mathematical learning, if a parent demonstrates elevated levels of math anxiety, their child is likely to be adversely affected by parental math anxiety, which results in a detrimental emotional experience (Haase et al., 2019).

As shown in Tables 7, 8, the negative association between parental involvement and the math anxiety of children was significant among parents with high math anxiety, but this association was non-significant among parents with low math anxiety. This counterintuitive finding is a key conclusion of this study. This result appears to contradict the conclusions of some prior research (e.g., Berkowitz et al., 2015; DiStefano et al., 2020; Schaeffer et al., 2018), which suggests that high-level parental math anxiety undermines the benefits of involvement. The discrepancy may be explained by differences in sample characteristics (Chinese vs. Western, parent-school meeting attendees), measurement (emotional/intellectual/behavioral management dimensions vs. global frequency) and child age (seventh grade vs. younger).

The findings reveal two coexisting pathways. First, parental math anxiety showed a positive association with the math anxiety of children (*r* = 0.29, *p* < 0.01). This is in line with emotional contagion theory (Hatfield et al., 1993) and previous research (Maloney et al., 2015; Vanbinst et al., 2020). This supports the view that children can “catch” math anxiety from their parents through daily interactions such as parents’ negative math-related talk, anxious body language and avoidance of math activities (DiStefano et al., 2020).

However, moderation analysis uncovered a second conditional pathway. Among parents with higher-level math anxiety, greater parental involvement was linked to lower-level math anxiety in their children. The counterintuitive finding suggests that highly math-anxious parents may interrupt or reverse the typical transmission of anxiety when choosing to positively engage. One explanation is an “awareness-compensation” mechanism: Because of their own negative experiences, these parents are possibly particularly motivated to adopt supportive, low-pressure strategies (e.g., rewarding effort and avoiding negative comments related to math) to protect their children from developing similar anxiety (Retanal et al., 2021). In contrast, parents with lower-level anxiety may not perceive math anxiety as a threat. Thus, their involvement lacks this targeted compensatory effort.

### 5.3 Gender differences in the moderating effect

In this study, whether the moderating effect of parental math anxiety differs by child gender was further examined. Separate hierarchical regressions for boys and girls validated that the interaction of parental involvement with parental math anxiety was significant only for girls (mother involvement × PMA: *B* = −0.139, *p* = 0.011; father involvement × PMA: *B* = −0.148, *p* = 0.026) rather than boys (both *p* > 0.05). This means that parental math anxiety only has a moderating effect on daughters. This may be because girls are more attuned to parents’ emotions and expectations in academic contexts, which makes them more sensitive to how parental math anxiety shapes the quality of involvement (Eccles, 1987). In contrast, the math anxiety of boys may be more influenced by peers, teachers or self-efficacy beliefs (Else-Quest et al., 2010). This gender-specific pattern is novel. It suggests that interventions targeting parental math anxiety might be especially effective for girls, though this remains to be tested in future intervention studies.

In the meantime, the results of this study align with ecosystem theory (Bronfenbrenner, 1979) and reveal a nuanced family dynamic: Notwithstanding the overall protective effect of parental involvement on the math anxiety of children, this effect was particularly pronounced when the level of parental math anxiety was high and non-significant when the level of parental math anxiety was low. This challenges the assumption that high parental math anxiety is uniformly detrimental and instead suggests that it may even enhance the benefits of involvement under certain conditions.

### 5.4 Theoretical and practical implications

From a theoretical perspective, a model examining the moderating effect of parental math anxiety on the association of parental involvement with the math anxiety of children was tested. The findings confirm and extend previous research (Barger et al., 2019; Maloney et al., 2015; Zhao et al., 2021; Si et al., 2022; Ching et al., 2021; Daches Cohen and Rubinsten, 2017; Del Río et al., 2017; Demirtaş and Uygun-Eryurt, 2020; Vanbinst et al., 2020), which provides empirical support for the idea that a dual effect (parental involvement and parental math anxiety) influences the math anxiety of children. To be specific, greater parental involvement is associated with reductions in the math anxiety of children (Zhao et al., 2021; Si et al., 2022; Ching et al., 2021), which highlights the positive potential of parents to actively participate in the education of their children.

However, consistent with emotional contagion theory (Hatfield et al., 1993), higher-level parental math anxiety is correlated with increased math anxiety in children (Vanbinst et al., 2020; DiStefano et al., 2020; Maloney et al., 2015). This implies that parental anxiety can be transmitted to children, which aggravates their anxiety and discomfort with math. This study highlights the important role of parental factors as a key component of the family environment that affects the math anxiety of children. These perspectives are integrated to provide a comprehensive framework offering insights into how parental involvement and parental math anxiety work together to influence the math anxiety of children.

From a practical perspective, this study provides empirical evidence for research on the math anxiety of children and theoretically expounds the complex association of parental involvement with the math anxiety of children in a new way. The results suggest that educators and parents should be concerned about parental math anxiety. In addition, the math anxiety of children can be effectively alleviated and their academic experience can be improved by promoting strategies to reduce parental math anxiety and encouraging positive, assertive parental involvement. In particular, creating a supportive, low anxiety learning environment is critical to mitigating the negative effects of parental anxiety.

### 5.5 Limitations and directions for future research

A few limitations exist in this study. First, a cross-sectional design was used, which thus led to the failure to draw causal inferences. Future longitudinal studies are warranted to determine the direction of the effects and examine how these relationships change over time.

Second, all data were gathered through self-report questionnaires. Harman’s one-way test indicated that this was not a major issue, but parental involvement was measured through the reports of children. Children may be unable to clearly distinguish between the involvement levels of their mothers and fathers, which may explain why the patterns for mothers and fathers are similar. Future studies should collect independent reports from each parent. Moreover, it would be preferable to include the observational measures of parent–child interactions during math study sessions.

Third, general academic anxiety, math achievement or the quality of parental involvement (e.g., the distinction between supportive and pressuring behaviors) was not measured. Consequently, it is unclear whether the observed effects are solely linked to math anxiety or whether the quality of parental involvement plays a role. These variables should be incorporated into future research to provide a more integrated understanding. Furthermore, the sample only comprised parents attending school meetings and completing the questionnaire, which may introduce selection bias.

Finally, a quantitative survey design was employed, which can reveal the overall relationships between variables. However, it is limited in capturing the underlying dynamics. Future research could use qualitative case studies to enhance quantitative findings, thereby providing a deeper understanding of the counterintuitive moderating effects identified in this study.

## 6 Conclusion

This study indicates that parental math anxiety moderates the relationship between parental involvement and the math anxiety of children. Despite the general negative correlation between parental involvement and the math anxiety of children (main effect), this association is more pronounced among parents with higher-level math anxiety. Conversely, the association is non-significant among parents with lower-level math anxiety. This counterintuitive pattern challenges the simplistic view that “high-level parental math anxiety is inevitably harmful”. Contrarily, the study suggests that parents with higher-level math anxiety may even enhance the protective effects of their involvement when actively involved. Furthermore, this moderating effect is particularly salient among girls. These findings contribute to a better understanding of the intergenerational transmission of math anxiety and provide more nuanced insights for family-based interventions.

## Data Availability

The raw data supporting the conclusions of this article will be made available by the authors, without undue reservation.
